# Portable, Low‐Field MRI for Alzheimer's Disease: Detecting Patterns of Atrophy Using Machine Learning Pipelines

**DOI:** 10.1002/alz70856_103075

**Published:** 2025-12-26

**Authors:** Ava Farnan, Annabel Sorby‐Adams, Jennifer Guo, Pablo Laso, Amanda Desenna, John Kirsch, Julia Zabinska, John R Dickson, Liliana A Ramirez Gomez, Pamela Shaefer, Seyedmehdi Payabvash, Adam de Havenon, Mattew Rosen, Kevin Sheth, Juan Eugenio Iglesias, Teresa Gomez‐Isla, W. Taylor Kimberly

**Affiliations:** ^1^ Massachusetts General Hospital and Harvard Medical School, Boston, MA, USA; ^2^ Athinoula A. Martinos Center for Biomedical Imaging, Boston, MA, USA; ^3^ Yale New Haven Hospital and Yale School of Medicine, New Haven, CT, USA; ^4^ Centre for Medical Image Computing, University College London, London, United Kingdom; ^5^ Computer Science and Artificial Intelligence Laboratory, Massachusetts Institute of Technology, Cambridge, MA, USA

## Abstract

**Background:**

Magnetic resonance imaging (MRI) can be used to monitor disease progression in Alzheimer's disease (AD). Portable, low‐field MRI (LF) facilitates point‐of‐care assessment and improves patient‐centered access. We developed a machine learning (ML) pipeline to use in conjunction with LF‐MRI to quantify brain morphometry and enable monitoring of AD patients in the outpatient clinic.

**Method:**

Patients with mild cognitive impairment (MCI) or dementia due to Alzheimer's disease (AD) were enrolled from the outpatient Massachusetts General Hospital (MGH) Memory Disorders Unit. A separate cohort of subjects with vascular comorbidities (VC) of comparable age were recruited from the Yale New Haven Hospital. LF‐MRI was acquired on a 0.064 T MRI (Hyperfine Inc), with conventional high‐field MRI (HF‐MRI; 1.5‐3 T) scans obtained within 7±11 months of LF‐MRI. T2 FLAIR sequences from HF and LF counterparts were processed through FreeSurfer‐based pipeline WMH‐SynthSeg for quantification of the following regions: whole brain, white matter, cortex, hippocampus, amygdala, putamen, pallidum, caudate, thalamus, accumbens, cerebellum, and ventricles.

**Result:**

The study population included MCI (*N* = 31, 72±7 years), AD (*N* = 24, 72±9 years), and VC (*N* = 22, 64±8 years). Correlations between HF and LF derived brain volumes was high across all brain regions (all *p* <0.05). The highest correlations were observed in the cortex and white matter (r=0.91; 95% CI 0.86, 0.94), lateral ventricle (r=0.98; 95% CI 0.97, 0.99), 3rd ventricle (r=0.95; 95% CI 0.92, 0.97), hippocampus (r=0.80; 95% CI 0.70, 0.87), caudate (r=0.82; 95% CI 0.73, 0.88), and amygdala (r=0.89; 95% CI 0.83, 0.93). The MCI and AD cohorts showed regional atrophy relative to the VC cohort (all regions *p* <0.05) except for the caudate and 4th ventricle (*p* >0.05). Regions with the most significant atrophy included the cortex, hippocampus, and amygdala (all *p* <0.001).

**Conclusion:**

LF‐MRI acquisition at the point‐of‐care for MCI or AD patients is feasible, and application of ML algorithms can generate brain volumes comparable to those derived from conventional counterparts, allowing for differentiation between VC and AD/MCI subgroups. Our findings demonstrate that LF‐MRI could be used as an accessible neuroimaging option for disease monitoring among those with cognitive impairment.

